# Incidence, Risk Factors and Consequences of Emergence Agitation in Adult Patients after Elective Craniotomy for Brain Tumor: A Prospective Cohort Study

**DOI:** 10.1371/journal.pone.0114239

**Published:** 2014-12-10

**Authors:** Lu Chen, Ming Xu, Gui-Yun Li, Wei-Xin Cai, Jian-Xin Zhou

**Affiliations:** Department of Critical Care Medicine, Beijing Tiantan Hospital, Capital Medical University, China National Clinical Research Center for Neurological Diseases, Beijing, 100050, China; Sun Yat-sen University, China

## Abstract

Emergence agitation is a frequent complication that can have serious consequences during recovery from general anesthesia. However, agitation has been poorly investigated in patients after craniotomy. In this prospective cohort study, adult patients were enrolled after elective craniotomy for brain tumor. The sedation-agitation scale was evaluated during the first 12 hours after surgery. Agitation developed in 35 of 123 patients (29%). Of the agitated patients, 28 (80%) were graded as very and dangerously agitated. By multivariate stepwise logistic regression analysis, independent predictors for agitation included male sex, history of long-term use of anti-depressant drugs or benzodiazepines, frontal approach of the operation, method and duration of anesthesia and presence of endotracheal intubation. Total intravenous anesthesia and balanced anesthesia with short duration were protective factors. Emergence agitation was associated with self-extubation (8.6% vs 0%, P = 0.005). Sedatives were administered more in agitated patients than non-agitated patients (85.7% vs 6.8%, P<0.001). In conclusion, emergence agitation was a frequent complication in patients after elective craniotomy for brain tumors. The clarification of risk factors could help to identify the high-risk patients, and then to facilitate the prevention and treatment of agitation. For patients undergoing craniotomy, greater attention should be paid to those receiving a frontal approach for craniotomy and those anesthetized under balanced anesthesia with long duration. More researches are warranted to elucidate whether total intravenous anesthesia could reduce the incidence of agitation after craniotomy.

**Trial Registration:**

ClinicalTrials.gov NCT00590499.

## Introduction

Emergence agitation is a significant clinical issue during recovery from general anesthesia [1]. The reported incidence varied from 3% to 21% in adult patients undergoing ear, nose and throat, ophthalmologic, abdominal, urologic, and vascular surgeries [2]–[5]. Emergence agitation can suddenly become dangerous and have serious consequences, such as self-extubation, accidental removal of catheters and injury. From our clinical experience, emergence agitation can occur in neurosurgical patients, and it is often difficult to manage. However, investigations in this subset of patients have been scarce. Patients after intracranial operations were excluded from three large cohort studies that investigated emergence agitation after general anesthesia [3]–[5]. Until now, only one observational study has explored the incidence of delirium in the intensive care unit (ICU) setting and included neurosurgical patients [6].

The causes of agitation are multifactorial. Studies in non-neurosurgical patients have identified several independent risk factors for emergence agitation, mainly including pain, endotracheal intubation, duration of surgery, and history of long-term treatment by anti-depressant agents [3]–[5]. Many of these risk factors are also frequent in patients after craniotomy, such as long duration of surgery, post-operative pain, and presence of endotracheal intubation [7], [8]. Moreover, it has not been investigated whether central nervous system diseases and intracranial manipulations would increase the incidence of emergence agitation. Based on these considerations, study is warranted to clarify the prevalence and risk factors of emergence agitation in neurosurgical population.

Patients after intracranial operations are more vulnerable to the stress resulted from emergence agitation during the recovery from general anesthesia [9], [10]. Physiological changes during agitation may cause intracranial hemorrhage and brain edema. Elevated oxygen consumption may disturb the brain oxygen demand-supply balance, and may result in ischemia ultimately. Several studies have demonstrated that these intracranial complications are correlated with adverse outcomes [11], [12]. However, the relationship between emergence agitation and intracranial complications is still poorly investigated.

In the present study, we prospectively enrolled adult patients after elective craniotomy for brain tumors, to investigate the incidence of emergence agitation, to identify the risk factors associated with agitation and to determine clinical outcomes. These results could provide basic data for the prevention and treatment of emergence agitation in post-operative neurosurgical patients.

## Materials and Methods

The protocol for this trial (in Chinese version, see S1 Protocol; in English version, see S2 Protocol) and supporting TREND Statement Checklist (see S1 Checklist) are available as supporting information.

### Ethics statement

The study was approved by the Institutional Review Board (IRB) of Beijing Tiantan Hospital, Capital Medical University (BJTTH-ICU-07-012). Because no attempts were made to change or influence standard clinical practices, informed consent was waived with IRB approval. The study protocol was registered on ClinicalTrials.gov (NCT00590499) (URL: http://clinicaltrials.gov/show/NCT00590499).

### Study setting and routine practice of post-operative care

We undertook this prospective study in a 12-bed neurosurgical ICU of a 1000-bed university hospital from July to December 2012. The nurse-to-bed ratio was 3∶1. During the study, routine practices of anesthesia and post-operative care were followed, and no attempts were made to change or influence the standard of care [13].

In our hospital, all intracranial operations are performed under general balanced anesthesia or total intravenous anesthesia (TIVA). Typically, anesthesia is induced with intravenous propofol and fentanyl or sufentanil. Tracheal intubation is facilitated with intravenous vecuronium or rocuronium. Anesthesia is maintained with propofol and/or sevoflurane, and fentanyl or sufentanil is administered intermittently, as needed. Vecuronium or rocuronium is administered according to train-of-four monitoring. The choice of agents is at the discretion of the anesthesiologist [13].

As long as there are enough beds, patients after craniotomy are routinely transferred directly to the neurosurgical ICU. However, the ICU can’t always provide overnight monitoring for every patient, and this occurs in about one day out of the five working days. In these cases, anesthesiologists and neurosurgeons discuss the status of the patient and decide whether he or she is suitable for return to the neurosurgical floor bed. Usually, patients without history of cardiovascular and respiratory illness, without pre- and intra-operative complications, and without delayed recovery from anesthesia, are transferred to the ward.

In the neurosurgical ICU, neurological examinations, including the Glasgow Coma Scale (GCS), focal signs and pupillary examination, are performed by nurses hourly or as needed. Hemodynamic and respiratory monitoring include 5-lead continuous electrocardiogram, pulse oximeter, noninvasive blood pressure and capnography. Post-operative computed tomography (CT) scans are not routinely obtained, although they are usually performed in patients exhibiting unexplained delayed awakening or new neurologic deficits. Patients are discharged from the neurosurgical ICU the next morning once their physiological statuses have stabilized, usually with normal neurological, hemodynamic and respiratory statuses.

The level of agitation and sedation is evaluated by Riker’s sedation-agitation scale (SAS), a 7-point scale ranging from unarousable (SAS = 1) to dangerous agitation (SAS = 7) (Table 1) [14]. We had incorporated the SAS into our clinical practice more than one year before the present study was undertaken. All of the physicians and nurses in the neurosurgical ICU were trained. Nurses assessed and documented the SAS of each patient every four hours or as needed. In patients with agitation (SAS = 5–7), both the nurse and physician performed careful evaluation to identify the possible organic causes of agitation, such as acute deterioration of the respiratory and circulatory systems, a new neurologic event, pain and hypoglycemia. Fentanyl (25 µg) was administered intravenously on an as-needed basis. Midazolam or propofol was used, and the level of sedation was titrated to an SAS score of 3 to 4.

**Table 1:** Riker’s sedation-agitation scale [14].

7	Dangerousagitation	Pulling at endotracheal tube, trying to remove catheters, climbing over bed rail, striking at staff, thrashing from side to side
6	Very agitated	Does not calm down despite frequent verbal reminders of limits, requires physical restraints, biting ET tube
5	Agitated	Anxious or mildly agitated, attempting to sit up, calms down with verbal instructions
4	Non-agitated	Calm and cooperative
3	Sedated	Calm, awakens easily, follows commands, difficult to arouse, awakens to verbal stimuli or gentle shaking but drifts off again, follows simple commands
2	Very sedated	Arouses to physical stimuli but does not communicate or follow commands, may move spontaneously
1	Unarousable	Minimal or no response to noxious stimuli, does not communicate or follow commands

### Definition and documentation of agitation

Agitation was defined as an SAS score of 5 to 7. During the study period, two investigators (GYL and WXC) evaluated and documented the SAS scores of the enrolled patients on an hourly basis. These investigators were chief nurses who did not participate in the care of the enrolled patients. Two other investigators (LC and MX) reviewed the nursing records daily. Finally, the maximal SAS for each patient was determined and confirmed by the four investigators in daily meetings.

### Study population

Consecutive patients admitted to the neurosurgical ICU after elective craniotomy under general anesthesia were screened on a daily basis. The inclusion criteria included both supratentorial and infratentorial intradural cranial operations for brain tumor. The exclusion criteria were age younger than 18 years old, emergency operations, unarousable state (SAS = 1) during the first 24 hours after the operation, and an interval longer than 24 hours between the end of the surgery and neurosurgical ICU admission. Patients were enrolled only once unless they were discharged from the hospital and re-admitted more than 180 days after the first enrollment.

SAS was evaluated for 12 hours after the operation. According to the maximal SAS score, the patients were divided into two groups: the agitation group (SAS = 5–7) and the non-agitation group (SAS≤4).

### Data collection

For each patient, the following data were collected at study entry: demographic characteristics (sex, age and body weight), history of smoking and alcohol abuse, long-term (>1 month) use of anti-depressant drugs or benzodiazepines, length of stay (LOS) in the hospital before the operation, and frontal location of the tumor. Data collected during the anesthesia and operation included: frontal approach of the operation, duration of anesthesia, amount of bleeding and anesthesia by TIVA. The post-operative data collected included: GCS at neurosurgical ICU admission, presence of endotracheal intubation, need for mechanical ventilation, presence of an external ventricular drainage (EVD) tube, complaints of pain, episodes of pulse oxygen saturation (SpO_2_) less than 90%, respiratory rate (RR) less than 8/minute, mean blood pressure (MAP) greater than 130 mm Hg or less than 70 mm Hg, and concentration of blood glucose greater than 10 mmol/L.

Patients were followed up for 72 hours after surgery. The primary outcome variables included: self-extubation of the endotracheal tube and accidental removal of central venous or bladder catheters. Secondary outcome variables included: unexpected re-operation within 72 hours after surgery and ICU discharge at post-operative day 1. The uses of sedatives (midazolam or propofol) and analgesics (fentanyl) during the ICU stay were also documented.

### Statistical analysis

Statistical analyses were performed using MS Excel for Mac (Microsoft Corporation, Beijing, China) and SPSS statistical software (version 20.0, SPSS, Chicago, IL, USA). Categorical variables are expressed as percentages. Continuous data were checked for normal distribution by the Kolmogorov-Smirnov test and are reported as the means and standard deviations (SDs) or the medians with 25th and 75th percentiles, when applicable.

The distribution of maximal SAS score was analyzed, and the incidence of agitation was calculated to present the epidemiologic characteristics. Univariate analyses between the agitation and non-agitation groups were performed. Categorical variables were analyzed by the χ^2^ test. Comparisons of continuous data were performed by using the unpaired t-test for normally distributed variables and the Mann-Whitney U test for non-normally distributed variables. Factors with P-values <0.20 were included in multivariate analysis (stepwise backward logistic regression) to identify independent predictors of agitation. Odds ratios and their 95% confidence intervals (CIs) were used to assess the independent contributions of significant factors. The Hosmer-Lemeshow test was used to determine the appropriateness of the model.

A P-value of less than 0.05 was considered statistically significant.

## Results

**Figure 1:**
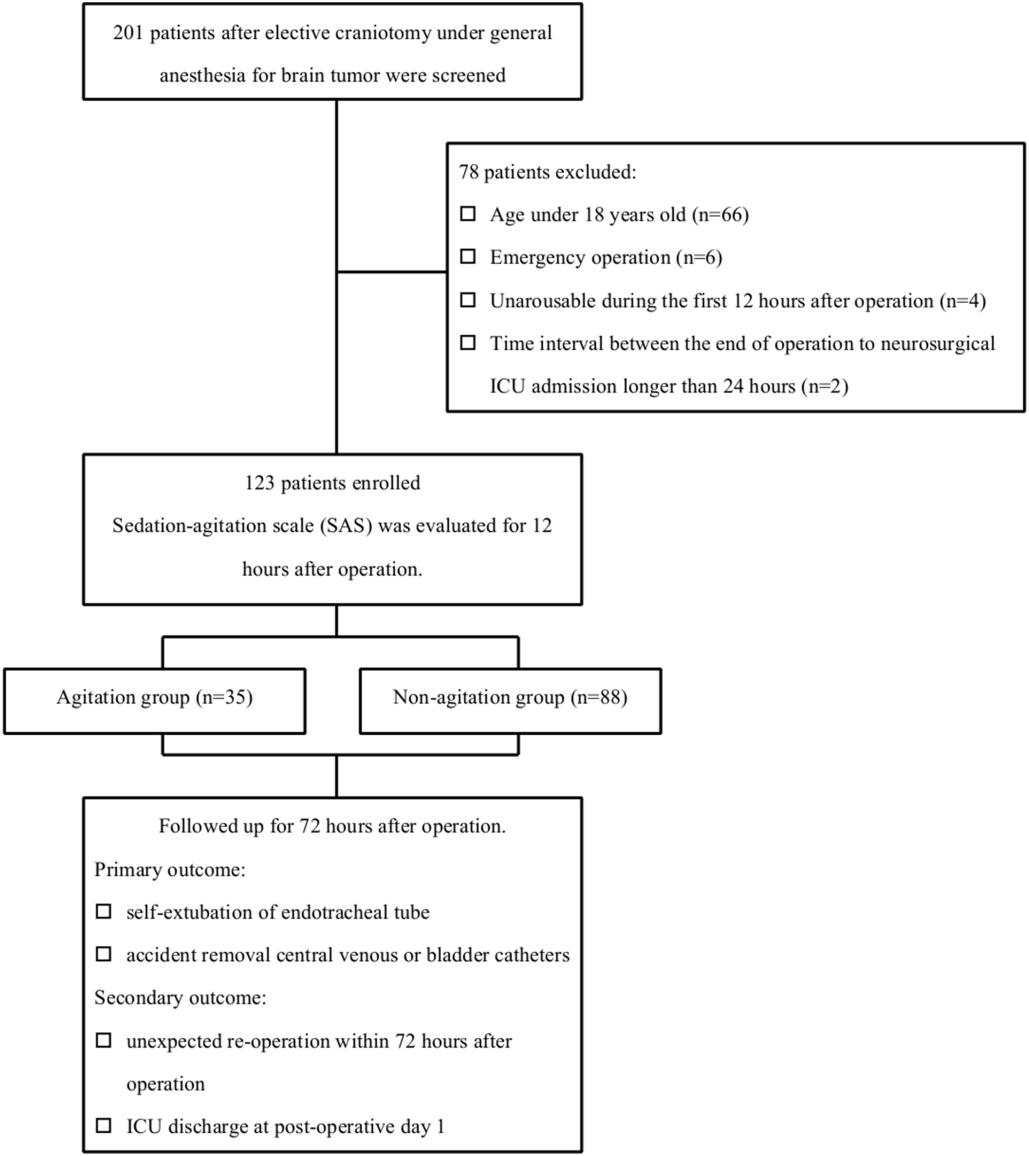
Patient flowchart.

**Figure 2:**
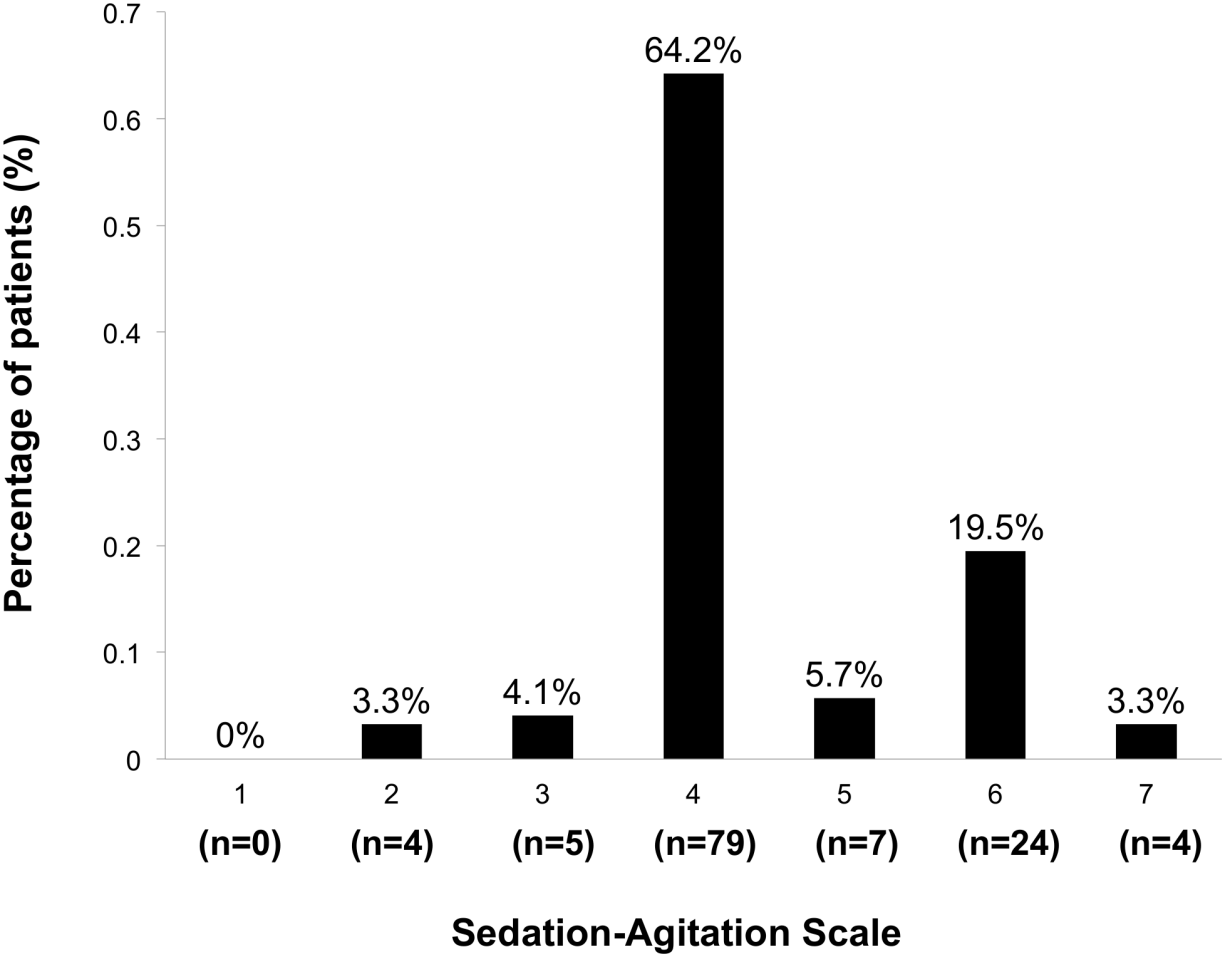
Distribution of the maximal sedation-agitation scale (SAS) in the study population.

The study did not deviate from the protocol. During the study period, 201 patients were admitted to the neurosurgical ICU after elective craniotomy under general anesthesia for brain tumor. Seventy-eight patients were excluded due to the following factors: age younger than 18 years old (n = 66), emergency operation (n = 6), unarousable during the first 12 hours after the operation (n = 4), and interval between the end of the operation and neurosurgical ICU admission longer than 24 hours (n = 2). In all, 123 patients were included in the analysis (Fig. 1 shows the patient flowchart). The mean age of the patients was 44±13 years old (20 to 68 years), and 45% were male. Agitation occurred in 29% of the patients (35 of 123 patients) during the first 12 hours after surgery. Fig. 2 shows the distribution of maximal SAS scores.

After the completion of the study, we retrospectively reviewed the documentation in operating room, and found that 17 patients after elective craniotomy for brain tumor were returned directly to the neurosurgical ward during the study period. The mean age of these patients was 36±6 years old (26 to 44 years), and 76% were male. Among these patients, tumors were located in frontal area in 3 (18%) patients, and approach of the operation were frontal in 5 (29%) patients.

### Risk factors

Univariate analysis showed that there were significant differences between the agitation and non-agitation groups in sex, history of long-term use of anti-depressant drugs or benzodiazepines, duration of anesthesia, amount of bleeding, anesthesia by TIVA, GCS at neurosurgical ICU admission and presence of endotracheal intubation (Table 2).

**Table 2:** Patient variables in the agitation and non-agitation groups.

Variables	Agitation (n = 35)	Non-agitation (n = 88)	P
Demographic data			
Male, n/N (%)	21/35 (60.0%)	34/88 (38.6%)	0.032
Age (yr, mean ± SD)	45±14	43±12	0.359
BW (kg, mean ± SD)	67±10	70±13	0.305
History			
Smoking, n/N (%)	9/35 (25.7%)	16/88 (18.2%)	0.349
Alcohol, n/N (%)	3/35 (8.6%)	8/88 (9.1%)	0.927
ADD or benzodiazepines, n/N (%)	4/35 (11.4%)	1/88 (1.1%)	0.009
Pre-operation			
LOS before surgery (day, mean ± SD)	5.1±2.6	5.2±2.5	0.786
Frontal location of the lesion, n/N (%)	1/35 (2.9%)	8/88 (9.1%)	0.231
Anesthesia and operation			
Frontal approach, n/N (%)	14/35 (40.0%)	24/88 (27.3%)	0.168
TIVA, n/N (%)*	1/35 (2.9%)	19/88 (21.6%)	0.011
Duration of anesthesia (hr, median [IQR])*	7.0 (5.7–8.3)	5.5 (4.0–6.5)	<0.001
AMT of bleeding (ml, median [IQR])#	500 (400–1000)	400 (200–600)	0.002
AMT of bleeding/hr (ml, median [IQR])#	80 (67–161)	76 (41–118)	0.072
Post-operation			
GCS at ICU admission (median [IQR])	3 (3–15)	15 (9–15)	<0.001
Endotracheal intubation, n/N (%)	17/35 (48.6%)	15/88 (17.0%)	<0.001
Mechanical ventilation, n/N (%)	3/35 (8.6%)	2/88 (2.3%)	0.110
Complaint of pain, n/N (%)	16/35 (45.7%)	47/88 (53.4%)	0.441
SpO_2_ <90%, n/N (%)	3/35 (8.6%)	2/88 (2.3%)	0.110
RR <8/min, n/N (%)	3/35 (8.6%)	2/88 (2.3%)	0.110
MAP>130 mm Hg, n/N (%)	3/35 (8.6%)	2/88 (2.3%)	0.110
MAP<70 mm Hg, n/N (%)	0/35 (0)	1/88 (1.1%)	0.527
BG >10 mmol/L, n/N (%)	5/35 (14.3%)	7/88 (8.0%)	0.286
EVD, n/N (%)	4/35 (11.4%)	7/88 (8.0%)	0.542

ADD: anti-depressant drug; AMT: amount; BG: blood glucose concentration; BW: body weight; EVD: external ventricular drainage; GCS: Glasgow Coma Scale; ICU: intensive care unit; IQR: inter-quartile range; LOS: length of stay; MAP: mean blood pressure; RR: respiratory rate; TIVA: total intravenous anesthesia; SpO_2_: pulse oxygen saturation.

*: Variable combined method and duration of anesthesia was used in the multivariate analysis.

# : Only amount of bleeding per hour was used in the multivariate analysis.

During the multivariable model building, a significant interaction was found between the duration of anesthesia and the method of anesthesia (TIVA or balanced anesthesia). The duration of anesthesia in patients anesthetized by TIVA (median [IQR]: 4.5 [3.0–6.4] hours) was significantly shorter than those receiving balanced anesthesia (6.0 [5.0–7.0] hours, P = 0.020). According to the median of duration of anesthesia in all 123 included patients (5.7 hours), we stratified patients to two clusters: short and long duration of anesthesia (≤5.7 hours vs >5.7 hours). In patients anesthetized by TIVA, no significant difference in the incidence of agitation was found between short and long duration of anesthesia (P = 0.350, Table 3). However, in patients receiving balanced anesthesia, the incidence of agitation was significantly lower in patients with short duration of anesthesia (18.0% vs 47.2%, P = 0.002, Table 3). So, we divided the included patients into three categories according to the method and duration of anesthesia: TIVA (category 1), balanced anesthesia with duration ≤5.7 hours (category 2) and balanced anesthesia with duration >5.7 hours (category 3). This combined variable of method and duration of anesthesia was used as a categorical covariate in the multivariate analysis, instead of separate variables of method and duration of anesthesia.

**Table 3:** Interaction between duration and method of anesthesia.

	Agitation (n = 35)	Non-agitation (n = 88)	P	Category
TIVA (n = 20)			0.350	
Duration of anesthesia ≤5.7 hours (n = 13)	0/13 (0%)	13/13 (100%)		1
Duration of anesthesia >5.7 hours (n = 7)	1/7 (14.3%)	6/7 (85.7%)		
Balanced anesthesia (n = 103)			0.002	
Duration of anesthesia ≤5.7 hours (n = 50)	9/50 (18.0%)	41/50 (82.0%)		2
Duration of anesthesia >5.7 hours (n = 53)	25/53 (47.2%)	28/53 (52.8%)		3

TIVA: total intravenous anesthesia.

Data in the table as expressed as n/N (%).

Patients were stratified to three categories according to the method and duration of anesthesia.

Multivariate analysis showed that male sex, history of long-term use of anti-depressant drugs or benzodiazepines, frontal approach of the operation, method and duration of anesthesia and presence of endotracheal intubation were independent predictors for agitation (Table 4). The Hosmer-Lemeshow test verified the validity of the model (P = 0.880).

**Table 4:** Independent predictors for agitation.

Independent risk factors	OR	95% CI	P
Male sex	4.5	1.5–13.4	0.007
History of long-term use of ADD or benzodiazepines	15.5	1.1–213.4	0.041
Frontal approach of the operation	3.7	1.2–12.0	0.027
Endotracheal intubation	7.7	2.5–24.3	<0.001
Method and duration of anesthesia			
Balanced anesthesia with duration >5.7 hours (category 3)	1 (reference)		0.018
Balanced anesthesia with duration ≤5.7 hours (category 2)	0.2	0.1–0.8	0.017
TIVA (category 1)	0.1	0.0–1.0	0.050

ADD: anti-depressant drug; CI: confidence interval; OR: odds ratio.

### Agitation-related outcomes

Table 5 shows the outcome variables. Agitation was associated with self-extubation of the endotracheal tube (8.6% vs 0%, P = 0.005). There was a significantly higher incidence of the use of sedatives in the agitation group (85.7% vs 6.8%, P<0.001).

**Table 5:** Outcome variables in the agitation and non-agitation groups.

Outcomes	Agitation (n = 35)	Non-agitation (n = 88)	P
Self-extubation, n/N (%)	3/35 (8.6%)	0/88 (0%)	0.005
Accident removal of catheters, n/N (%)	1/35 (2.9%)	1/88 (1.1%)	0.496
Use of sedatives, n/N (%)	30/35 (85.7%)	6/88 (6.8%)	<0.001
Use of analgesics, n/N (%)	16/35 (45.7%)	47/88 (53.4%)	0.441
Unexpected re-operation, n/N (%)	2/35 (5.7%)	1/88 (1.1%)	0.138
ICU discharge at POD1, n/N (%)	32/35 (91.4%)	84/88 (95.5%)	0.358

ICU: intensive care unit; POD1: post-operative day 1.

## Discussion

The present study showed that emergence agitation was common in patients after elective craniotomy for brain tumor performed under general anesthesia. Of the total included patients, 29% suffered at least one episode of agitation during the first 12 hours after surgery. Moreover, of all of the agitated patients, 80% were graded as very or dangerously agitated (Fig. 1); in these cases, analgesics and sedatives were needed. Published data have shown that the incidence of emergence agitation has varied from 3% to 21% in patients undergoing non-neurosurgical operations [2]–[5]. Lepouse et al. reported that the incidence of emergence agitation was 4.7% in the post-anesthesia care unit (PACU), using Riker’s SAS as the evaluation instrument [3]. Radtke et al. investigated the prevalence of delirium in non-intubated adult patients after general anesthesia [4]. The Richmond Agitation-Sedation Scale (RASS) was used to identify emergence delirium (agitation, RASS≥+1) and hypoactive emergence (RASS≤–2), and 5% of the enrolled patients exhibited agitation [4]. In another observational study with 2000 post-operative patients, a simple three-point scale was used to assess agitation, and the incidences of mild, moderate and severe agitation were reported to be 10.6%, 8.9% and 1.8%, respectively [5]. However, according to this scale, only grades of moderate and severe agitation were comparable to agitation determined by SAS or RASS. Emergence agitation has seldom been reported in neurosurgical populations. In van den Boogaard et al.’s study, delirium was assessed in ICU patients using the Confusion Assessment Method-ICU (CAM-ICU) [6]. Delirium was divided into three subtypes: hyperactive, hypoactive and mixed. This study included 74 neurosurgical patients and found that the incidences of hyperactive, hypoactive and mixed delirium were 0%, 5.4% and 4.1%, respectively. Until now, there has not been a standard instrument for the evaluation of emergence agitation after general anesthesia. Several sedation and agitation scales have been developed to measure the depth of sedation and to identify agitation in critically ill patients, such as the SAS, Motor Activity Assessment Scale, RASS, etc. [15]. In 2013, the revised Society for Critical Care Medicine (SCCM) clinical practice guidelines for the management of pain, agitation, and delirium suggested that the RASS and SAS were the most valid and reliable assessment tools for detecting agitation and for measuring the quality and depth of sedation in adult ICU patients [16]. In the present study, we chose the SAS to evaluate agitation. The major advantages of the SAS are its precise grading of agitation and ease of use (Table 1). Although no studies have been performed to investigate the reliability and validity of the SAS in the evaluation of agitation in patients after craniotomy, we believe that it is comparable to using it in ICU patients. Our results indicated that agitation was a frequent complication in patients after craniotomy and that most patients were severely agitated. To the best of our knowledge, this was the largest study to investigate emergence agitation in patients after elective craniotomy under general anesthesia.

We identified five independent predictors for emergence agitation after craniotomy under general anesthesia (Table 4). Similar to the results from the non-neurosurgical population, male sex, long-term (>1 month) use of anti-depressant drugs or benzodiazepines, and presence of endotracheal intubation were associated with agitation [3]–[5]. Previous studies showed that the duration of surgery [3] and the anesthetic technique [5] might be risk factors for emergence agitation. Agitation seems more likely to occur after long duration of surgery and general anesthesia with inhalational agents. Several randomized, controlled trials have compared the rate of agitation between various inhalational anesthetics and propofol in non-neurosurgical adult patients [17]–[19]. The results of these studies have indicated that propofol might decrease the incidence of emergence agitation compared with inhalational anesthetics. In our study, we identified the method and duration of anesthesia as a combined predictor for emergence agitation in patients after craniotomy. Balanced anesthesia with long duration was associated with significantly increased emergence agitation (47.2%, Table 3), whereas TIVA and short duration of balanced anesthesia were manifested as protective factors (Table 4). In a multicenter, randomized, controlled trial conducted in Europe, adult patients were enrolled after elective supratentorial intracranial surgery under general anesthesia, and emergence agitation was compared among three different anesthesia maintenance methods (sevoflurane-remifentanil, sevoflurane-fentanyl and propofol-remifentanil) [20]. No significant differences in agitation were found among the three groups. However, incidences of agitation reported in the study (3.7% to 6.5%) were much lower than those in our study. Lower incidence of agitation in this study might be contributed to the relatively short duration of surgery (294 to 318 minutes). There was no formal standard for the use of TIVA in our institute during the study period, and the choice of anesthesia method was at the discretion of the anesthesiologist. We consulted several attending anesthesiologists, and found that TIVA was usually chosen for operations with anticipated short duration and less sophistication. Further investigation is needed to clarify the influence of anesthetic technique, especially TIVA, on emergence agitation in neurosurgical patients.

Interestingly, we also found that a frontal approach for the operation was an independent risk factor for emergence agitation (Table 4). Because the executive function of the frontal lobes involves cognitive and emotional behaviors, damage during a frontal approach for craniotomy could result in abnormal behaviors [21]. The relationship between frontal lobe lesions and agitation in patients with traumatic brain injury and Alzheimer’s disease has been studied [22], [23]. Until now, no studies have been performed to investigate the relationship between the approach for craniotomy and emergence agitation. However, we cannot explain why a frontal tumor location did not increase the incidence of agitation. Indeed, in the patients in the non-agitation group, the tumor was located in frontal lobes more often than in the agitation group (Table 2).

Pain has been identified as an independent risk factor for emergence agitation in non-neurosurgical patients [4], [5]. However, we found no significant differences in the incidence of patient’s compliant of pain and the use of fentanyl between the agitation and non-agitation groups (Table 2 and Table 5). Acute pain is common after craniotomy [8]. By using the patient’s self-report pain scales, such as visual analogue scale (VAS) or verbal numerical rating scale (NRS), several studies have found that 37% to 63% of patients undergoing craniotomy experience moderate to severe pain during the first postoperative day [24]–[26]. In our study, 51% (63/123) of patients complained of pain during the study, and this was comparable to the previous reports. Evaluations of self-report pain scales require patient’s ability to communication. Consciousness impairment due to intracranial manipulation and post-operative sedation may influence the reliability of VAS or NRS evaluations. For these reasons, we did not incorporate the evaluations of self-report pain scales into our clinical practice, and only documented the patient’s complaint of pain. However, recent study indicated that NRS could be reliably assessed in the neurologically critically ill [27]. Yu et al. enrolled 151 adult patients in neurological, neurosurgical, neurosciences or surgical trauma ICUs from three hospitals, and found that reliable NRS score could be obtained in 70% of the evaluations [27]. In our study population, the majority of patients were not very sedated (96.7% with SAS≥3, Fig. 2), and this also indicated that the evaluation of self-report pain scale might not impossible in patients after craniotomy. We have planed to modify our strategy of pain evaluation and analgesia to incorporate VAS and behavioral pain scales into our clinical routine.

We found that agitation resulted in a high rate of self-extubation and sedative use (Table 5). Studies of PACU patients did not report the consequences of emergence agitation. In contrast, it has been established that agitation is associated with complications in ICU patients, particularly in those receiving mechanical ventilation [28]. Woods et al. investigated severe agitation among medical ICU patients and found that agitated patients were more likely to self-extubate and had longer ICU stays and more mechanical ventilation days [29]. Jaber et al.’s study in medical-surgical ICU patients yielded similar results [30]. We did not find that emergence agitation was associated with unplanned re-operation or the length of ICU stay for post-operative monitoring. Because complaints of pain were comparable in the agitation and non-agitation groups, there was no difference in the use of analgesics between the two groups (Table 5).

There were several limitations of our study. First, we only enrolled ICU admitted patients after elective craniotomy. In clinical situation in our institute, ICU beds were not always available to this population, and this might result in that the only high-risk patients with agitation were included in the study. After study completion, we retrospectively analyzed the documentation in operating room, and found that only 7.8% (17 in 218) of patients after elective craniotomy for brain tumor were transferred to neurosurgical ward during the study period. Incidence of emergence agitation was still high (25%) even if adding these returned-to-ward patients to the denominator. However, this limited the generalization of our results to the whole population of patients after craniotomy for brain tumors. Additionally, we only enrolled patients after elective craniotomy for brain tumors. Therefore, the results of the present study cannot be applied to other patient populations, for example, brain injured patients with trauma, stroke and ischemic brain injury. Second, we only reported the complaint of pain, instead of the evaluation of pain scores (such as VAS or NRS). As mentioned above, acute pain is common after craniotomy. This may underestimate the incidence of pain in the study population. Third, previous study has found that hypoactive delirium might be more common than agitation in patients after general anesthesia [4], [6]. In present study, we did not measure the hypoactive delirium. However, we found that GCS at ICU admission was lower in agitation group (Table 2). During recovery from general anesthesia, brain function remains inhibited to some degree [31]. In this condition, some patients might react excessively to external stimuli, and emergence agitation can occur suddenly, resulting in serious consequences. In patients undergoing craniotomy, it is important to differentiate the cause of impaired consciousness among the delay of anesthesia recovery, intracranial deficits and hypoactive delirium. Physicians should pay greater attention to the process of recovery from general anesthesia.

## Conclusions

In conclusion, emergence agitation was a frequent complication in patients after elective craniotomy for brain tumors. Nearly 30% of the patients exhibited at least one episode of agitation during the early post-operative period, and most of these patients were severely agitated. Emergence agitation was associated with the risk of self-extubation. Independent predictors for agitation included male sex, history of long-term use of anti-depressant drugs or benzodiazepines, frontal approach of the operation, method and duration of anesthesia and presence of endotracheal intubation. TIVA and balanced anesthesia with short duration were protective factors. The clarification of risk factors could help to identify the high-risk patients, and then to facilitate the prevention and treatment of agitation. For patients undergoing craniotomy, greater attention should be paid to those receiving a frontal approach for craniotomy and those anesthetized under balanced anesthesia with long duration. More researches are warranted to elucidate whether TIVA could reduce the incidence of agitation after craniotomy.

## Supporting Information

S1 Checklist **The supporting TREND Statement Checklist.**
(PDF)

S1 Protocol **The protocol for this trial in Chinese version.**
(PDF)

S2 Protocol **The protocol for this trial in English version.**
(PDF)

## References

[1] Whitlock EL, Vannucci A, Avidan MS (2011) Postoperative delirium. Minerva Anestesiol 77:448–456. 21483389 PMC3615670

[2] Rose DK (1996) Recovery room problems or problems in the PACU. Can J Anaesth 43:R116–128. 8706214 10.1007/BF03011674

[3] Lepouse C, Lautner CA, Liu L, Gomis P, Leon A (2006) Emergence delirium in adults in the post-anaesthesia care unit. Br J Anaesth 96:747–753. 16670111 10.1093/bja/ael094

[4] Radtke FM, Franck M, Hagemann L, Seeling M, Wernecke KD, et al (2010) Risk factors for inadequate emergence after anesthesia: emergence delirium and hypoactive emergence. Minerva Anestesiol 76:394–403. 20473252

[5] Yu D, Chai W, Sun X, Yao L (2010) Emergence agitation in adults: risk factors in 2,000 patients. Can J Anaesth 57:843–888. 20526708 10.1007/s12630-010-9338-9

[6] van den Boogaard M, Schoonhoven L, van der Hoeven JG, van Achterberg T, Pickkers P (2012) Incidence and short-term consequences of delirium in critically ill patients: A prospective observational cohort study. Int J Nurs Stud 49:775–783. 22197051 10.1016/j.ijnurstu.2011.11.016

[7] Himmelseher S, Pfenninger E (2001) Anaesthetic management of neurosurgical patients. Curr Opin Anaesthesiol 14:483–490. 17019134 10.1097/00001503-200110000-00004

[8] Flexman AM, Ng JL, Gelb AW (2010) Acute and chronic pain following craniotomy. Curr Opin Anaesthesiol 23:551–557. 20717011 10.1097/ACO.0b013e32833e15b9

[9] Bruder N, Ravussin P (1999) Recovery from anesthesia and postoperative extubation of neurosurgical patients: a review. J Neurosurg Anesthesiol 11:282–293. 10527148 10.1097/00008506-199910000-00009

[10] Bruder NJ (2002) Awakening management after neurosurgery for intracranial tumours. Curr Opin Anaesthesiol 15:477–482. 17019241 10.1097/00001503-200210000-00001

[11] Manninen P, Raman S, Boyle K, EL-Beheiry H (1999) Early postoperative complications following neurosurgical procedures. Can J Anaesth 46:7–14. 10078396 10.1007/BF03012507

[12] Sawaya R, Hammoud M, Schoppa D, Hess KR, Wu SZ, et al (1998) Neurosurgical outcomes in a modern series of 400 craniotomies for treatment of parenchymal tumors. Neurosurgery 42:1044–1056. 9588549 10.1097/00006123-199805000-00054

[13] Cai YH, Zeng HY, Shi ZH, Shen J, Lei YN, et al (2013) Factors influencing delayed extubation after infratentorial craniotomy for tumour resection: a prospective cohort study of 800 patients in a Chinese neurosurgical centre. J Int Med Res 41:208–217. 23569147 10.1177/0300060513475964

[14] Riker RR, Picard JT, Fraser GL (1999) Prospective evaluation of the sedation-agitation scale for adult critically ill patients. Crit Care Med 27:1325–1329. 10446827 10.1097/00003246-199907000-00022

[15] Sessler CN, Grap MJ, Ramsay MA (2008) Evaluating and monitoring analgesia and sedation in the intensive care unit. Crit Care 12 (Suppl 3)S2. 10.1186/cc6148 PMC2391268 18495053

[16] Barr J, Fraser GL, Puntillo K, Ely EW, Gelinas C, et al (2013) Clinical practice guidelines for the management of pain, agitation, and delirium in adult patients in the intensive care unit. Crit Care Med 41:263–306. 23269131 10.1097/CCM.0b013e3182783b72

[17] Kim YS, Chae YK, Choi YS, Min JH, Ahn SW, et al (2012) A comparative study of emergence agitation between sevoflurane and propofol anesthesia in adults after closed reduction of nasal bone fracture. Korean J Anesthesiol 63:48–53. 22870365 10.4097/kjae.2012.63.1.48 PMC3408515

[18] Liang C, Ding M, Du F, Cang J, Xue Z (2014) Sevoflurane/propofol coadministration provides better recovery than sevoflurane in combined general/epidural anesthesia: a randomized clinical trial. J Anesth Feb 21, Epub ahead of print. 10.1007/s00540-014-1803-0 24557087

[19] Liu GY, Chen ZQ, Zhang ZW (2014) Comparative study of emergence agitation between isoflurane and propofol anesthesia in adults after closed reduction of distal radius fracture. Genet Mol Res 13: Epub ahead of print. 10.4238/2014.January.24.9 24615079

[20] Citerio G, Pesenti A, Latini R, Masson S, Barlera S, et al (2012) A multicentre, randomised, open-label, controlled trial evaluating equivalence of inhalational and intravenous anaesthesia during elective craniotomy. Eur J Anaesthesiol 29:371–379. 22569025 10.1097/EJA.0b013e32835422db

[21] Potegal M (2012) Temporal and frontal lobe initiation and regulation of the top-down escalation of anger and aggression. Behav Brain Res 231:386–395. 22085875 10.1016/j.bbr.2011.10.049

[22] Vonder Haar C, Friend DM, Mudd DB, Smith JS (2013) Successive bilateral frontal controlled cortical impact injuries show behavioral savings. Behav Brain Res 240:153–159. 23201357 10.1016/j.bbr.2012.11.029

[23] Senanarong V, Cummings JL, Fairbanks L, Masterman DM, O’Connor SM, et al (2004) Agitation in Alzheimer's disease is a manifestation of frontal lobe dysfunction. Dement Geriatr Cogn Disord 17:14–20. 14560060 10.1159/000074080

[24] Gottschalk A, Berkow LC, Stevens RD, Mirski M, Thompson RE, et al (2007) Prospective evaluation of pain and analgesic use following major elective intracranial surgery. J Neurosurg 106:210–216. 17410701 10.3171/jns.2007.106.2.210

[25] Mordhorst C, Latz B, Kerz T, Wisser G, Schmidt A, et al (2010) Prospective assessment of postoperative pain after craniotomy. J Neurosurg Anesthesiol 22:202–206. 20479664 10.1097/ANA.0b013e3181df0600

[26] Nair S, Rajshekhar V (2011) Evaluation of pain following supratentorial craniotomy. Br J Neurosurg 25:100–103. 21158514 10.3109/02688697.2010.534199

[27] Yu A, Teitelbaum J, Scott J, Gesin G, Russell B, et al (2013) Evaluating pain, sedation, and delirium in the neurologically critically ill-feasibility and reliability of standardized tools: a multi-institutional study. Crit Care Med 41:2002–2007. 23863231 10.1097/CCM.0b013e31828e96c0

[28] Tate JA, Devito Dabbs A, Hoffman LA, Milbrandt E, Happ MB (2012) Anxiety and agitation in mechanically ventilated patients. Qual Health Res 22:157–173. 21908706 10.1177/1049732311421616 PMC3598123

[29] Woods JC, Mion LC, Connor JT, Viray F, Jahan L, et al (2004) Severe agitation among ventilated medical intensive care unit patients: frequency, characteristics and outcomes. Intensive Care Med 30:1066–1072. 14966671 10.1007/s00134-004-2193-9

[30] Jaber S, Chanques G, Altairac C, Sebbane M, Vergne C, et al (2005) A prospective study of agitation in a medical-surgical ICU: incidence, risk factors, and outcomes. Chest 128:2749–2757. 16236951 10.1378/chest.128.4.2749

[31] Silverstein JH, Timberger M, Reich DL, Uysal S (2007) Central nervous system dysfunction after noncardiac surgery and anesthesia in the elderly. Anesthesiology 106:622–628. 17325520 10.1097/00000542-200703000-00026

