# Supplementary material for: Incidence, Risk Factors and Consequences of Emergence Agitation in Adult Patients after Elective Craniotomy for Brain Tumor: A Prospective Cohort Study
Source: PLoS One. 2014 Dec 10;9(12):e114239. doi: 10.1371/journal.pone.0114239 (PMC4262354; doi:10.1371/journal.pone.0114239)
Supplement: S1 Protocol — The protocol for this trial in Chinese version. (PDF) [file pone.0114239.s002.pdf]

[伦理审查申报材料]

开颅术后患者躁动的前瞻性研究：

发生率、危险因素和转归

（2011 年 第一版）

首都医科大学附属北京天坛医院重症医学科

2011 年 10 月

目　录

一、研究计划书

二、病例报告表

三、研究者简历

四、伦理审查申请表

一、研究计划书

研究计划书

开颅术后患者躁动的前瞻性研究：

发生率、危险因素和转归

（2011 年 第一版）

首都医科大学附属北京天坛医院重症医学科

2011 年 10 月

背景

躁动是重症医学热点研究问题之一。有文献报道了急性躁动在内科重症加强医疗病房（ICU）、外科 ICU 和术后恢复室（PACU）患者中的发生率、危险因素和对转归的影响[1-4]。这些研究的结果表明，躁动导致不良转归，包括意外拔管、医院获得性感染、ICU 和住院时间延长。临床经验表明，急性躁动也经常发生于开颅术后患者，且同样会导致不良转归，特别是术后颅内出血[5,6]。然而，到目前为止，对于开颅术后患者的急性躁动，相关研究并不充分。本研究前瞻性纳入开颅术后患者，调查急性躁动的发生率、危险因素及对转归的影响。

研究计划

一、研究设计

前瞻性队列研究，不干扰和改变现有临床诊疗常规。

二、实施单位

首都医科大学附属北京天坛医院神经外科 ICU。

三、患者纳入

连续筛查平诊全麻开颅手术后进入 ICU 接受监测治疗的成年患者。

排除标准包括：

1. 年龄小于 18 岁；
2. 急诊手术；
3. 72 小时内再次手术；
4. 术后 24 小时内昏迷；
5. 手术结束到进入 ICU 超过 24 小时。

四、躁动的判定

应用 Riker 躁动-镇静量表（SAS）评估患者（附表）[7]。

由 2 名不参与临床护理的护士长每小时评估患者 SAS。2 名研究者复习患者 SAS 评分。这 4 名研究者每天讨论患者 SAS 状况，做出决定。

根据患者术后 12 小时内最高 SAS 评分将患者分为 2 组，躁动组为 SAS 5-7 分，

非躁动组为SAS 1-4分。

附表：SAS量表

7	Dangerous agitation	Pulling at ET tube, trying to remove catheters, climbing over bed rail, striking at staff, thrashing side-to-side
6	Very agitated	Does not calm, despite frequent verbal reminding of limits; requires physical restraints, biting ET tube
5	Agitated	Anxious or mildly agitated, attempting to sit up, calms down to verbal instructions
4	Non-agitated	Calm and cooperative
3	Sedated	Calm, awakens easily, follows commands Difficult to arouse, awakens to verbal stimuli or gentle shaking but drifts off again, follows simple commands
2	Very sedated	Arouses to physical stimuli but does not communicate or follow commands may move spontaneously
1	Unarousable	Minimal or no response to noxious stimuli, does not communicate or follow commands

五、危险因素的收集

（一）术前资料：人口学资料（性别、年龄、体重）、吸烟和饮酒史、抗抑郁药或苯二氮卓类药物服用史（超过1个月）、术前住院时间、肿瘤位于额部；

（二）术中资料：额部手术入路、麻醉时间、术中出血量、全凭静脉麻醉（TIVA）。

（三）术后资料：入ICU时格拉斯哥昏迷量表（GCS）、保留气管插管、需要机械通气、脑室外引流管、主诉疼痛、指尖氧饱和度（SpO2）低于90%、呼吸频率低于8次/分、平均动脉压高于130 mmHg或低于70 mmHg、血糖低于10 mmol/L。

六、转归评价

随访患者至出院。

主要转归指标包括：自行拔除气管插管、意外拔除其他导管。

次要转归指标包括：ICU 停留时间、出院时格拉斯哥转归评分（GOS）。

统计学处理

计数资料采用百分比描述，计量资料首先进行正态性检验，正态分布的计量资料用均数±标准差表示，非正态分布的计量资料采用中位数（四分位数）描述，根据 SAS 评分将患者分为躁动组（SAS 5-7 分）和非躁动组（SAS 1-4 分）。计数资料采用卡方检验或 Fisher 精确检验，正态分布计量资料采用 Student T 检验，非正态分布计量资料采用 Mann-Whitney U 检验。然后将单因素分析 P＜0.20 的变量引入多因素 logistic 回归分析以确定影响延迟拔管的独立影响因素。所有资料采用 SPSS16.0 统计学软件分析。统计学检验水平为 P<0.05。

研究进度及样本量

研究预计于 2012 年 7 月至 12 月实施，计划纳入样本量 120 例。

免除知情同意书申请

由于本研究不改变现行临床诊疗常规，申请免除签署知情同意书。

参考文献

1. Woods JC, Mion LC, Connor JT, et al. Severe agitation among ventilated medical intensive care unit patients: frequency, characteristics and outcomes. Intensive Care Med 2004; 30:1066-1072
2. Jaber S, Chanques G, Altairac C, et al. A prospective study of agitation in a medical-surgical ICU: incidence, risk factors, and outcomes. Chest 2005; 128:2749-2757
3. Lepouse C, Lautner CA, Liu L, et al. Emergence delirium in adults in the post-anaesthesia care unit. Br J Anaesth 2006; 96:747-753
4. Radtke FM, Franck M, Hagemann L, et al. Risk factors for inadequate emergence after anesthesia: emergence delirium and hypoactive emergence. Minerva Anestesiol 2010; 76:394-403
5. Manninen PH, Raman SK, Boyle K, et al. Early postoperative complications following neurosurgical procedures. Can J Anaesth 1999; 46:7-14
6. Sutcliffe AJ. Neuroanaesthesia: key points during the perioperative period. Minerva Anestesiol 2003; 69:739-750
7. Riker RR, Picard JT, Fraser GL. Prospective evaluation of the Sedation-Agitation Scale for adult critically ill patients. Crit Care Med 1999; 27:1325-1329

二、病例报告表

项目名称

开颅术后患者躁动的前瞻性研究：发生率、危险因素和转归

病例报告表

(Case Report Form)

姓名缩写：__________

住 院 号：___________

CRF 号：___________

入组日期： 年 月 日

承担单位：北京天坛医院神经外科 ICU

版本号：第一版 修订日期：2011.10

填 表 说 明

1. 所有病例观察表必须由所在中心项目负责人指定人员填写。缩写数据无比真实，应于原始数据资料（即病历）保持一致。
2. 用墨蓝色或黑色圆珠笔或中性笔填写。
3. 病历报告表填写应准确、清晰、不得随意涂改，错误之处纠正时需用横线居中划出，并签署修改姓名拼写缩写及修改时间。
4. 所有选择项目的□内用√标注。
5. 住院号：按照该患者实际住院号填写。
6. 为保护患者隐私，患者姓名不能出现在 CRF 表上。患者姓名缩写四格，根据患者的姓名每个字的首字母靠左边填写；二字姓名填写二字首字母、三字姓名填写三字首字母；四字姓名填写复姓第一个字的首字母。

基本资料

**入选标准：**患者必须符合以下所有标准方可入选研究	是	否
1. 年龄>18 岁；	□	□
2. 平诊开颅手术后入住 ICU。	□	□
**排除标准：**有以下任何特征的患者不能进入研究		
1. 年龄小于 18 岁；	□	□
2. 急诊手术；	□	□
3. 72 小时内再次手术；	□	□
4. 术后 24 小时内昏迷；	□	□
5. 手术结束到进入 ICU 超过 24 小时。	□	□

一般情况	
姓名缩写：	□□□□
病案号：	□□□□□□□□
ICU 床号：	□□□□
性别：	□1 男　□2 女
年龄：	____岁
身高：	_______cm
体重：	____kg
科室：	__________

手术前情况			
抗抑郁药服用史	□1 是	□2 否	药物名称_______服用时间______
苯二氮卓类药物服用史	□1 是	□2 否	药物名称_______服用时间______
吸烟史	□1 是	□2 否	如果有吸烟史，______年，______支/天
饮酒史	□1 是	□2 否	如果有饮酒史，______年，______两/天
肿瘤部位	□1 额部	□2 非额部	
术前住院时间	______天		

手术中情况		
额部手术入路	□1 是	□2 否
全凭静脉麻醉	□1 是	□2 否
麻醉时间	______分钟	
术中出血量	______ml	

入 ICU 时情况		
GCS	E_____M____V_____	
气管插管	□1 是	□2 否
机械通气	□1 是	□2 否
脑室外引流管	□1 是	□2 否

ICU 监测资料

	时间 hour														
项目	0	1	2	3	4	5	6	7	8	9	10	11	12		
RR bpm															
BP mmHg															
SpO_2 %															
BG mmol/L															
SAS															
GCS E/V/M	/ /	/ /	/ /	/ /	/ /	/ /	/ /	/ /	/ /	/ /	/ /	/ /	/ /	/ /	/ /
咪达唑仑															
丙泊酚															
芬太尼															
意外 ET															
意外其他 T															

SAS 评价标准

7	危险躁动	拔管、跨越床栏、辗转翻身、攻击行为
6	非常躁动	无法平静、语言无法劝解、咬气管插管、需要物理约束
5	躁动	烦躁、轻度躁动、试图坐起、语言劝阻有效
4	无躁动	平静合作
3	镇静	平静易唤醒、遵嘱、对声音有反应
2	非常镇静	物理刺激可唤醒、不遵嘱、可表现为自主运动
1	无法唤醒	对疼痛刺激无反应或反应不足

转归资料

术后第一天
术后第一天是否脱机
术后第一天是否拔管　　☐1 是　☐2 否
术后第一天是否转出　　☐1 是　☐2 否
术后第一天 GCS 评分　　E___V___M___

术后 72 小时
术后 72 小时是否转出　　☐1 是　☐2 否
术后 72 小时是否再手术　☐1 是　☐2 否

出院
GOS 评分
☐　1 死亡
☐　2 植物状态
☐　3 重残（生活需要他人辅助）
☐　4 轻残（生活自理）
☐　5 恢复良好

观察者签字　____________________
日期　　　　_____年____月____日

三、研究者简历

首都医科大学附属北京天坛医院医学伦理委员会
IRB of Beijing Tiantan Hospital Affiliated to Capital Medical University

研究者简历

Resume of Investigator

姓名	周建新		
联系方式	67098019		
现任职务：	首都医科大学附属北京天坛医院副院长	职 称	主任医师，教授，博士生导师

单位名称：首都医科大学附属北京天坛医院重症医疗科
地址（邮编）：天坛西里 6 号，100050

教育背景：

1985.08-1990.07 首都医科大学 医学生

1995.08-2000.03 中国协和医科大学 硕士、博士研究生

工作经历：

1990.08-1995.07 首都医科大学附属复兴医院 住院医师

2000.04-2002.02 四川大学华西医院 博士后

2002.03-今 首都医科大学附属北京天坛医院 ICU 主任、副院长

主要临床研究经验：

吸入麻醉药作用机理的研究：发表 SCI 论著 4 篇

神经危重患者的镇痛镇静研究：发表 SCI4 篇，注册临床 RCT 研究 2 项

参加 GCP 培训经历：
否

专业协会或组织（最相关的）：
中国医师协会重症医学医师分会副会长、北京医学会危重症医学分会常委
中国医师协会神经外科医师分会神经重症专家委员会主任委员

发表文章（与研究相关的）：

1. Zhou JX, Luo NF, Liang XM, Liu J: The efficacy and safety of intravenous emul isoflurane in rats. Anesthesia and analgesia 2006, 102(1):129-134.
2. Wang Q, Zheng Y, Lu J, Chen L, Wang GN, Zhou JX: Isoflurane potency in mic the first and second parity. J Am Assoc Lab Anim Sci 2009, 48(6):714-717.
3. Wang Q, Zheng Y, Lu J, Chen L, Wang J, Zhou JX: Selective breeding of mice s with different sensitivity to isoflurane. Chinese medical journal 2010, 123(10):1315-1319.

研究者签名：[illegible]　　　　日期：2011.12.1

四、伦理审查申请表

首都医科大学附属北京天坛医院医学伦理委员会

IRB of Beijing Tiantan Hospital Affiliated to Capital Medical University

伦理审查申请表

Ethical Review Application Form

**一、项目概况**	
项目名称	开颅术后患者急性躁动的发生率、危险因素及转归的临床研究
申办者	首都医科大学附属北京天坛医院重症医学科
临床研究组长单位	首都医科大学附属北京天坛医院
临床研究参加单位	
我院承担科室	首都医科大学附属北京天坛医院重症医学科
我院主要研究者	周建新
我院研究者与研究助理	周建新、陈律
**二、研究摘要**	
研究设计(可多选)	□病例对照研究 ■队列研究 □横断面研究 □非随机对照研究 □随机对照研究 □应用盲法 □其他：
研究背景和目的	对于神经外科术后患者，躁动发生率和危险因素，相关研究不足
样本量、统计分析	纳入 120 例患者，根据术后 12 小时 SAS 评分分为躁动组和非躁动组，进行危险因素和转归的统计学分析
拟研究时间	2012 年 7 月 1 日 至 2012 年 12 月 31 日
**三、受试者招募、费用和补偿**	
谁负责招募（可多选）	■ 项目负责人 ■ 研究者 □ 医生 □ 护士 □ 其他：________
使用招募材料	■否 □是 →请提交审查
与研究有关的医疗检查与治疗	■免费 □部分免费 □不免费
**四、主要研究者责任声明**	我将遵循我国法律法规和国际伦理准则以及伦理委员会的要求，开展本项研究：<br>● 在审查批件失效期前提交跟踪审查申请报告；<br>● 进行方案修改时要报告伦理委员会，获得批准后执行（注：为避免对受试者紧急伤害的修正方案等可以先执行，然后及时报告）；<br>● 及时报告严重不良事件和影响风险与受益比的非预期不良事件；<br>● 及时报告提前终止研究、或其他伦理委员会的重要决定；<br>● 随时应伦理委员会的要求，报告正在进行的研究的有关信息；<br>● 提交最后的结题报告。
主要研究者签字	[illegible]
日期	2011 年 12 月 1 日
