# Supplementary material for: Incidence, Risk Factors and Consequences of Emergence Agitation in Adult Patients after Elective Craniotomy for Brain Tumor: A Prospective Cohort Study
Source: PLoS One. 2014 Dec 10;9(12):e114239. doi: 10.1371/journal.pone.0114239 (PMC4262354; doi:10.1371/journal.pone.0114239)
Supplement: S2 Protocol — The protocol for this trial in English version. (PDF) [file pone.0114239.s003.pdf]

STUDY PROTOCOL

A Prospective Study of Agitation in Post-craniotomy Patients:

Incidence, Risk factors, and Outcomes

Department of Critical Care Medicine,

Beijing Tiantan Hospital, Capital Medical University

October, 2011

Background

Agitation has been investigated in patients admitted to medical intensive care unit (ICU), surgical ICU and post-anesthesia care unit (PACU) [1-4]. Results of these studies indicate that agitation is associated with worse outcome, such as unplanned catheter removal, nosocomial infections, prolonged ICU and hospital stay, and mortality. Clinical experience shows that agitation occurs in patients after craniotomy, and this might result in serious consequences, especially for post-operative hematoma [5,6]. But up to now, agitation in post-operative neurosurgical patients is poorly studied. In present study, we prospectively investigated acute post-operative agitation in the neuro-ICU after craniotomy. The objective of this study was to define the incidence, risk factors and outcome of acute agitation in post-operative neurosurgical patients.

Study protocol

I Study design

Prospective cohort study. No attempt will be made to change or influence the standard practice of patient care.

II Setting

12-bed Neurosurgical ICU in an University Affiliated Hospital.

III Enrollment

All consecutive admitted patients after an elective craniotomy under general anesthesia will be included.

Exclusion criteria:

1. age under 18 years old;
2. emergency operation;
3. re-operation within 72 hours
4. unarousable during the first 24 hours after operation;
5. time interval between the end of operation to neurosurgical ICU admission longer than 24 hours.

IV Judgment of agitation

The Riker sedation-agitated scale (SAS) [7] will be employed to assess each patient's level of agitation (Table).

Two chief nurses, who will not involve the patient care, evaluated and

documented the SAS of enrolled patient hourly. Two investigators will review nursing records daily. The maximal SAS for each patient will be determined and confirmed by these four investigators in daily meeting.

According to the maximal SAS in the first 12 hours after operation, patients will be divided into 2 categories: non-agitated patients (Riker SAS levels 1-4) and agitated patients (Riker SAS levels 5-7).

Riker sedation-agitation scale

7	Dangerous agitation	Pulling at ET tube, trying to remove catheters, climbing over bed rail, striking at staff, thrashing side-to-side
6	Very agitated	Does not calm, despite frequent verbal reminding of limits; requires physical restraints, biting ET tube
5	Agitated	Anxious or mildly agitated, attempting to sit up, calms down to verbal instructions
4	Non-agitated	Calm and cooperative
3	Sedated	Calm, awakens easily, follows commands Difficult to arouse, awakens to verbal stimuli or gentle shaking but drifts off again, follows simple commands
2	Very sedated	Arouses to physical stimuli but does not communicate or follow commands may move spontaneously
1	Unarousable	Minimal or no response to noxious stimuli, does not communicate or follow commands

V Risk factors

Collection of potential risk factors include:

1. Pre-operative data: demographic characteristics (gender, age and body weight); history of smoke and alcohol abuse, long-term (>1 month) use of anti-depressive drugs or benzodiazepines; length of stay (LOS) in hospital before operation; frontal location of the tumor.
2. Data during anesthesia and operation included: frontal approach of the operation, duration of anesthesia, amount of bleeding and anesthesia by total intravenous anesthesia (TIVA).

Post-operative data included: Glasgow coma scale (GCS) at neurosurgical ICU admission, presence of endotracheal intubation, need mechanical ventilation, presence of external ventricular drainage (EVD) tube, complaint of pain, episode of pulse oxygen saturation (SpO_2) below 90%, respiratory rate (RR) below 8/minute, mean blood pressure (MAP) above 130 mmHg or below 70 mmHg, and concentration of blood glucose above 10 mmol/L.

VI Outcomes

Patients will be followed up to hospital discharge.

Primary outcomes include: self-extubation of endotracheal tube and accident removal central venous or bladder catheters.

Secondary outcomes include: length of stay in ICU and Glasgow outcome score (GOS) at hospital discharge.

Statistical analysis

Categorical variables are expressed as percentages. Continuous data are checked for normal distribution by Kolmogorov-Smirnov test, and are shown as mean and standard deviation (SD) or median with the 25th and 75th percentiles, when applicable.

Distribution of maximal SAS will be analyzed and incidence of agitation will be calculated to present the epidemiology knowledge. Univariate analyses between the agitation and non-agitation groups are carried out. Categorical variables are analyzed by χ^2 test. Comparisons of continuous data are performed by using unpaired t-test for normally distributed variables, and the Mann-Whitney U test for non-normally distributed variables. Factors with a P-value < 0.20 will be included in multivariate analysis (stepwise backward logistic regression) to identify the independent factors of agitation. Odds ratios and their 95% confidence intervals (CIs) are used to assess the independent contribution of significant factors. The Hosmer and Lemeshow test will be used to determine appropriateness of the model.

A P-value of less than 0·05 is considered statistically significant.

Planing schedule and sample

Study will be carried out in July, 2012, and is anticipated to finish in December, 2012. We plan to enroll 120 patients.

References

1. Woods JC, Mion LC, Connor JT, et al. Severe agitation among ventilated medical intensive care unit patients: frequency, characteristics and outcomes. Intensive Care Med 2004; 30:1066-1072
2. Jaber S, Chanques G, Altairac C, et al. A prospective study of agitation in a medical-surgical ICU: incidence, risk factors, and outcomes. Chest 2005; 128:2749-2757
3. Lepouse C, Lautner CA, Liu L, et al. Emergence delirium in adults in the post-anaesthesia care unit. Br J Anaesth 2006; 96:747-753
4. Radtke FM, Franck M, Hagemann L, et al. Risk factors for inadequate emergence after anesthesia: emergence delirium and hypoactive emergence. Minerva Anestesiol 2010; 76:394-403
5. Manninen PH, Raman SK, Boyle K, et al. Early postoperative complications following neurosurgical procedures. Can J Anaesth 1999; 46:7-14
6. Sutcliffe AJ. Neuroanaesthesia: key points during the perioperative period. Minerva Anestesiol 2003; 69:739-750
7. Riker RR, Picard JT, Fraser GL. Prospective evaluation of the Sedation-Agitation Scale for adult critically ill patients. Crit Care Med 1999; 27:1325-1329
